# Melatonin alleviates heat stress-induced testicular damage in dairy goats by inhibiting the PI3K/AKT signaling pathway

**DOI:** 10.1007/s44154-022-00068-9

**Published:** 2022-11-14

**Authors:** Yundie Liu, Hui Cai, Xinrui Guo, Aili Aierken, Jinlian Hua, Baohua Ma, Sha Peng

**Affiliations:** College of Veterinary Medicine, Shaanxi Centre of Stem Cells Engineering & Technology, Northwest Agriculture and Forestry University of Science and Technology, Yangling, 712100 Shaanxi China

**Keywords:** Natural heat stress, Melatonin, PI3K/AKT signaling pathway, Spermatocytes, Apoptosis

## Abstract

Current measures mainly focus on how melatonin reduces physiological heat stress in animals, but its effects on reproductive damage to male dairy goats have been neglected. This study aimed to determine the protective effect of melatonin on male reproduction during heat stress in dairy goats and to further explore its mechanisms. A natural heat stress model of Saanen dairy goats was used to assess testicular tissue damage 7 days after heat stress and to examine semen quality changes during a spermatogenic cycle. RNA-seq, Western blot, RT–qPCR, and immunofluorescence staining were used to explore the mechanism by which melatonin protects against heat stress-induced reproductive damage and to validate the results. The data suggested that melatonin significantly alleviated the heat stress-induced decrease in sperm quality, protected varicose tubule structure, reduced the levels of heat shock proteins and apoptotic proteins and protected the spermatocytes and round spermatozoa, which are mainly affected by heat stress. RNA-seq results suggest that melatonin inhibits the PI3K/AKT signaling pathway, reduces the level of p-AKT, and promotes elevated BCL-2. In addition, melatonin treatment could upregulate the gene expression of MT2 which was downregulated by heat stress and improve the change in extracellular matrix components and restore serum testosterone levels. Our results suggest that melatonin can protect against testicular and spermatogenic cell damage and improve semen quality in male dairy goats under heat stress. This study provides an important reference for subsequent studies on the molecular mechanisms of melatonin in protecting male reproductive processes under heat stress and using exogenous melatonin to prevent heat stress.

**Supplementary Information:**

The online version contains supplementary material available at 10.1007/s44154-022-00068-9.

## Introduction

Heat stress (HS) during summer is becoming more common due to global warming. In livestock farming, the mating period of goats is short and occurs close to the summer. The low semen quality in male animals caused by high temperatures seriously affects mating efficiency and economic benefits.

High temperature induces the apoptosis of germ cells mainly by inducing the accumulation of excessive reactive oxygen species (ROS) in cells; these ROS disrupt cell physiology (Tao W, 2022), attack lipoproteins, and damage mRNA and proteins (Huang Y, 2022). Some germ cells avoid apoptosis, and excessive ROS levels can damage and fragment the DNA in these cells (Guo H, 2021). Some researchers found that heat treatment inhibits the repair of the DNA double-stranded breaks that occur during homologous recombination, and this effect is mediated through H4K16ac deacetylation (Chakraborty S, 2022). In addition, studies have shown that the immune responses in organisms are extremely sensitive to DNA damage. A small number of DNA fragments can activate the innate immune response; this response can be described as stress-induced “germline DNA damage-induced systemic stress resistance”, which is also known as sterile autoinflammation caused by tissue damage (Ermolaeva MA, 2013). Both the series of intracellular responses by ROS and the sterile inflammation caused by DNA fragmentation, there are numerous studies suggesting the involvement of PI3K/AKT signaling pathway (Wang X, 2022; Datta S, 2022). Oxidative stress-related processes interfere with androgen receptor pathways in testicular cells (Somasekharan SP, 2022); in contrast, the levels of androgens in individual animals have significant positive impacts on resistance to acute stress reactions (Handa RJ, 2022).

Melatonin (MT), which is as a hypothalamic neuroendocrine hormone whose receptors are widely distributed in various tissues and organs, is involved in stress responses, immune regulation (Baekelandt S, 2019), and the maintenance of steroid hormone levels (Li Y, 2015). Melatonin and its derivatives have been reported to activate the ROS scavenging system to exert its antioxidant effects (Yu JC, 2022; Hwang SJ, 2021). Research suggests that melatonin, which is an indirect antioxidant, can directly bind to ROS radicals and inhibit lipid peroxidation reactions by inhibiting pro-oxidant enzyme activities (endothelial nitric oxide synthase activity) (Aridas JDS, 2018) and increasing antioxidant enzyme expression (glutathione peroxidase levels) (Janjetovic Z, 2014). Melatonin could occur via activation of Nrf2-mediated PI3K/AKT signaling pathway (Janjetovic Z, 2017), stimulates the synthesis and activation of ROS scavenging enzymes and other antioxidants, and promotes DNA repair (Sevilla A, 2022). Considering the role of melatonin in regulating reproduction, its antioxidant effects, and its unique biosafety profile (Barchas J, 1967), we hypothesized that melatonin protects against heat stress in the testis.

Therefore, investigating the mechanism underlying the impaired spermatogenesis in male animals caused by HS as well as the possible positive effects of melatonin could be of great importance to livestock farming. This experiment informed the development of production practices that prevent the impairment of male reproductive performance caused by HS.

## Results

### Heat stress-induced decrease in semen quality and the effect of melatonin treatment on this process

**Fig. 1:**
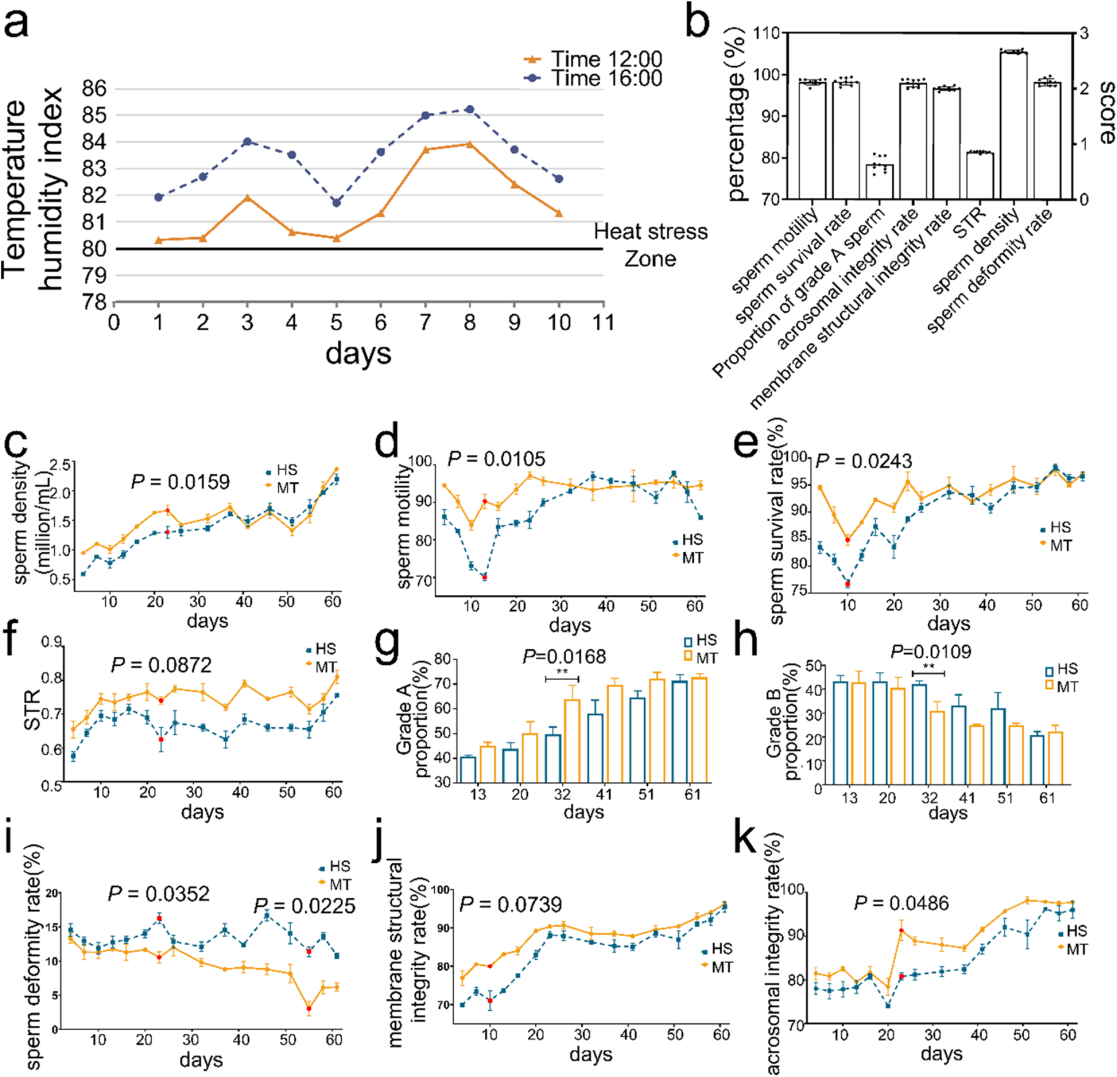
Low semen quality due to heat stress and the effects of melatonin on it. **a** THI index. **b** Indices of the NC group. **c** Sperm density in the HS vs. MT groups. **d** Sperm motility in the HS vs. MT groups. **e** sperm viability in the HS vs. MT groups. **f** STR in the HS vs. MT groups. **g** The percentage of class A sperm in the HS vs. MT groups. **h** The percentage of class B sperm in the HS vs. MT groups. **i** The deformity rate in the HS vs. MT groups. **j** The rate of plasma membrane sperm in the HS vs. MT groups. **k** The rate of acrosome intact sperm in the HS vs. MT group. ^*^P < 0.05; ^**^P < 0.01

First, we determined the effect of natural heat stress modeling during the modeling period by assessing the temperature humidity index (THI). The THI remained above the 80 range during the ten-day modeling period (Table S3, Fig. 1a), indicating that the dairy goats had suffered a heat-stressed environment and that the natural heat stress model was successfully established. Then, we detected semen quality once every 3 days for 61 days after mimicry to determine the effect of heat stress and melatonin treatment on semen quality. Semen samples were collected from male dairy goats of the same breed in autumn as a natural control (NC) group (Fig. 1b). The results of dynamic indices suggested that heat stress caused a significant decrease in sperm density, viability, and survival rate in dairy goats compared to the NC group (Fig. 1b) (Fig. 1c; d; e). The lowest point occurred on days 10–13 after experimental treatment.

Further analysis of the sperm dynamic indices showed that the percentage of both average path straight-line separation (STR) and effective sperm count (i.e., grade A sperm) decreased significantly in the heat stress (HS) group (Fig. 1f; g). The STR visually indicates the ability of sperm to move forward, while the effective sperm rate directly reflects the percentage of sperm with the ability to fertilize. Among them, we found that spermatozoa of grade A in the melatonin treatment (MT) group showed a significant rebound on day 32; spermatozoa of class B, i.e., typical heat-stressed spermatozoa, decreased significantly on day 32 after modeling compared to the HS group (Fig. 1g; h). These results indicate that melatonin can promote the repair process of semen heat damage and return it to the level of the NC group more quickly. To investigate how heat stress affected the dynamic parameters of spermatozoa, we performed morphological tests using sperm smears. The results showed that heat stress significantly increased the sperm malformation rate (Fig. 1i) and decreased membrane structure and acrosome integrity (Fig. 1j; k). Additionally, the MT group had a lower sperm malformation rate and significantly improved both sperm membrane structure and acrosome integrity.

These results indicate that heat stress significantly increased the percentage of malformed spermatozoa and damaged sperm membrane integrity and acrosome structure, which ultimately led to a significant decrease in the rate of effective spermatozoa and continued to impair semen quality and fertilization ability. The thermal damage caused by heat stress does not disappear immediately when the heat stops but has a certain lag and persistence, with subsequent effects beyond one spermatogenic cycle. Additionally, melatonin pretreatment protected the spermatozoa to a certain extent, stabilized the structure of sperm, and promoted the repair of sperm structure after the damage of heat stress, restoring the semen quality to the NC group level more quickly.

### Heat stress-induced testicular damage and spermatogenic apoptosis, and the effect of melatonin treatment on this process

**Fig. 2:**
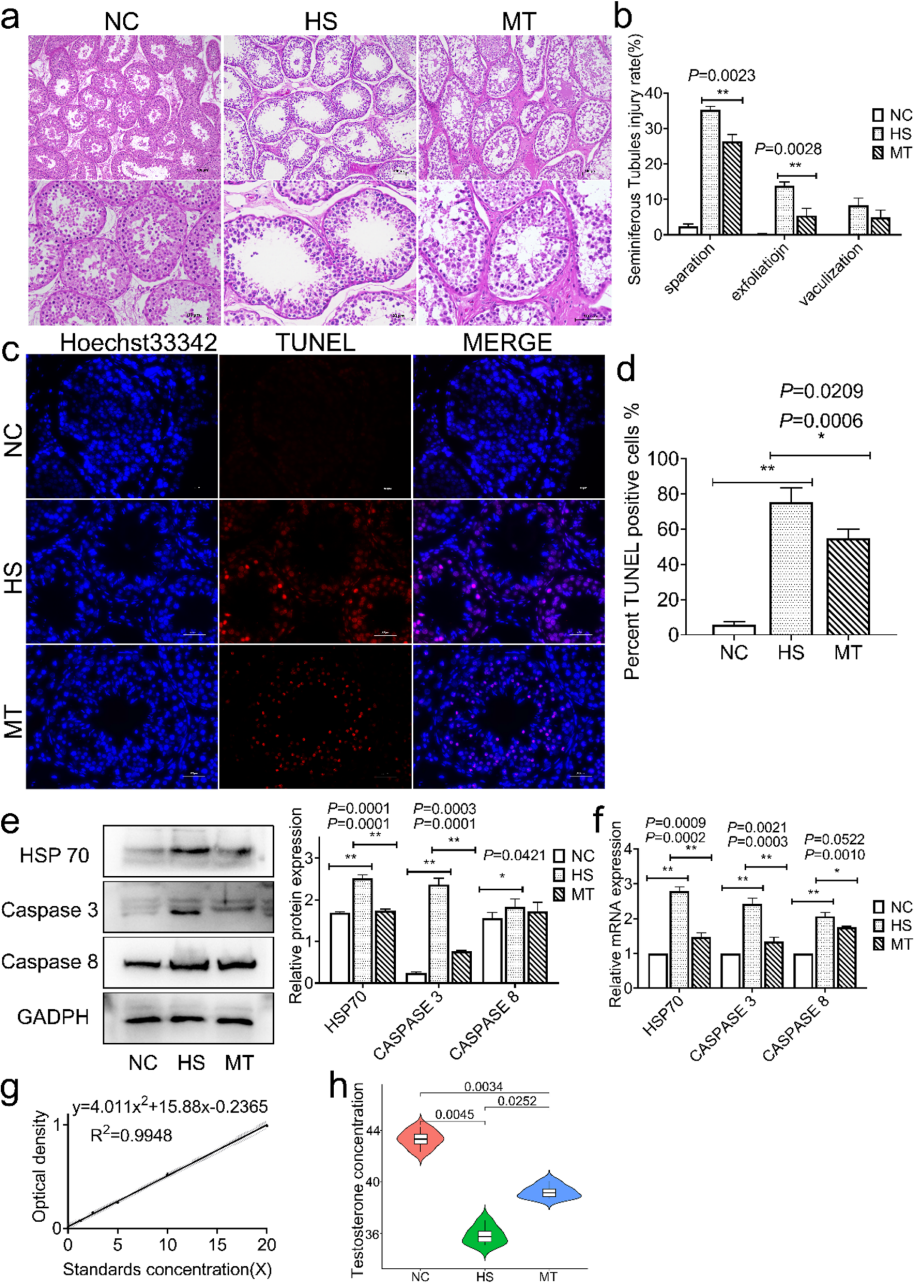
Heat stress-induced testicular tissue damage and spermatogenic cell apoptosis, inhibition of serum testosterone levels and the effect of melatonin on them. **a** Representative tissue H&E-stained light micrographs (Scale bar: 100 μm). **b** The percentage of spermatogenic tubules in the three types of damage (isolated, detached, vacuolated). **c** TUNEL assay for germ cell apoptosis. Blue fluorescence, localization of Hoechst 33342 dye; red fluorescence, TUNEL+ signal (Scale bar: 300 μm). **d** TUNEL staining analysis, the positive mean rate in the field of view. **e.** Left. HSP70, Caspase 3, and Caspase 8 protein expression; Right. Grayscale value quantification. **f** HSP70, Caspase 3, and Caspase 8 mRNA expression quantification. **g** Serum testosterone ELISA standard curve. R^2^ = 0.9948. **h** ELISA of serum testosterone levels. Mean grayscale values quantify relative expression. ^*^P < 0.05; ^**^P < 0.01

To explain how heat stress affects the spermatogenesis process at the tissue structure level, we collected testes from dairy goats on day 7 after experimental treatment. H&E staining suggested that heat exposure caused structural damage to testicular tissue, disruption of the spermatogenic tubule structure, disturbed arrangement of cell layers, and reduced the number of layers (Fig. 2a). Then, we found that melatonin treatment significantly reduced the percentage of lumenal separation and detachment of spermatogenic tubules by statistically classifying this damage (Fig. 2b) and alleviating varicocele thermal damage. Then, we found a significant apoptotic response to heat stress with DNA breakage damage by performing TUNEL staining (Fig. 2c). Simultaneously, the number of TUNEL-positive cells was significantly decreased in the MT group compared to the HS group (Fig. 2d).

The results of the tissue protein assay using Western blotting suggested that the levels of heat shock protein HSP70, apoptotic protein Caspase 3, and Caspase 8 were significantly increased (Fig. 2e), indicating that heat damage and apoptotic response in testis tissue continued over 7 days after heat stress treatment. Data from the MT group showed that melatonin significantly reduced the protein and mRNA levels of HSP70 and Caspase 3 (Fi. 2f). In addition, considering the ability of melatonin to act on the gonadal axis, we measured serum testosterone levels in dairy goats. The results showed that melatonin significantly elevated serum testosterone levels in response to heat stress inhibition (Fig. 2g; h). The above results showed that heat treatment caused significant local damage to the varicocele in the testis; apoptosis and DNA damage remained substantial 7 days after heat cessation and significantly reduced the number of spermatogenic cells. In addition, the effects of melatonin treatment consisted of a significant reduction in the expression levels of molecules associated with testicular heat injury and an increase in suppressed serum testosterone levels.

### Heat stress mainly affects spermatocytes and round spermatocytes in the testis

**Fig. 3:**
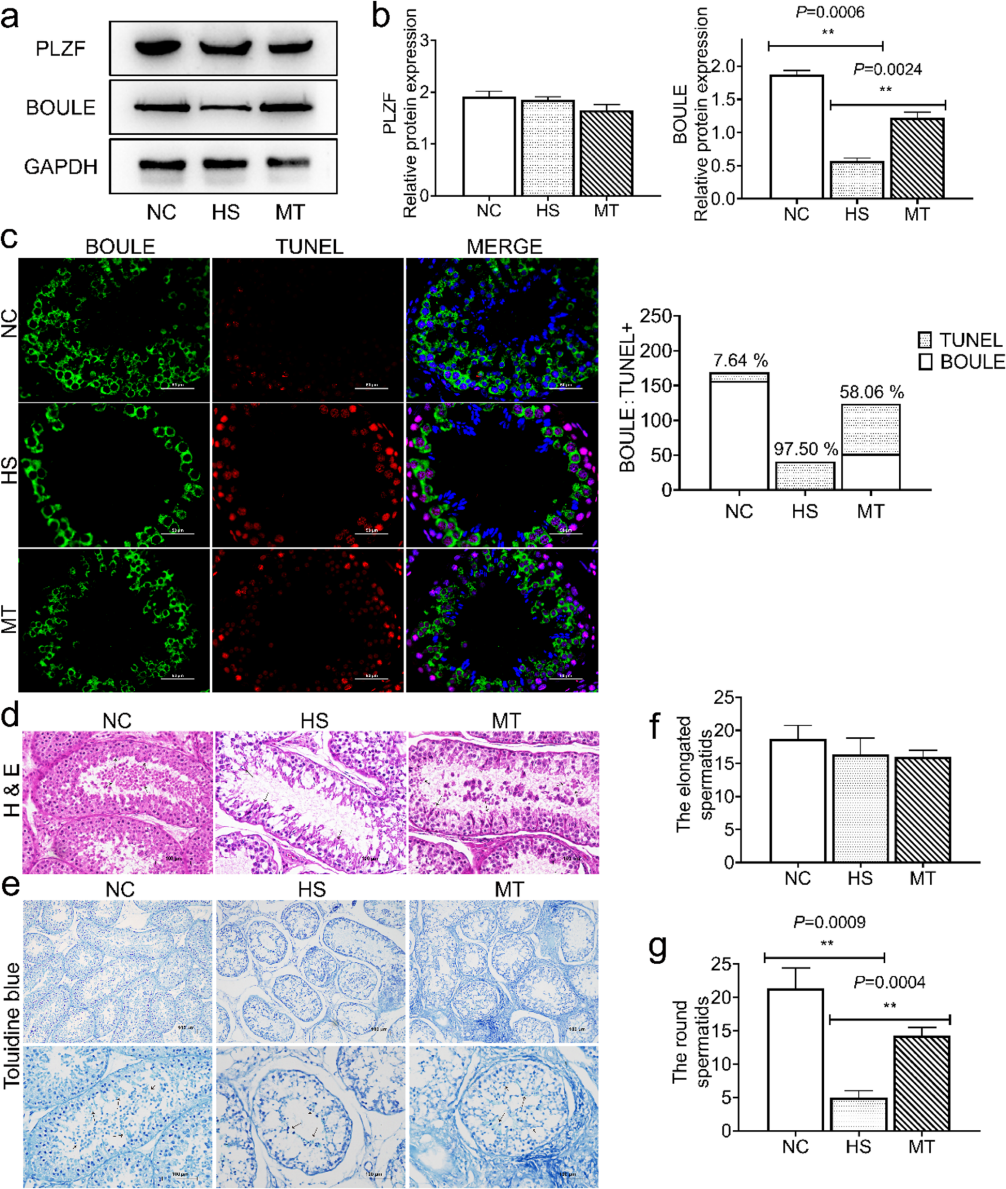
Heat stress mainly affects the number of spermatocytes and round spermatozoa in spermatogenic cells. **a** PLZF and BOULE protein expression. **b** Left. Grayscale value quantification of PLZF; Right. Grayscale value quantification of BOULE. **c** Left. Confocal microscopy for BOULE and TUNEL immunofluorescence co-staining. Blue fluorescence, localization of DAPI dye; green fluorescence, BOULE protein localization; red fluorescence, TUNEL+ signal; Right. BOULE and TUNEL+ count (Scale bar: 50 μm). **d** Representative tissue H&E-stained light micrographs of three representative groups (Scale bar: 100 μm). Short-tailed arrows indicate round spermatozoa, and long-tailed arrows indicate elongated spermatozoa. **e** Representative toluidine blue-stained micrographs of three representative groups (Scale bar: 100 μm). Short-tailed arrows indicate round spermatozoa, and long-tailed arrows indicate elongated spermatozoa. **f** Counts of elongated spermatozoa from toluidine blue staining results. **g** Counts of the round spermatozoa from toluidine blue staining results. Mean grayscale values quantifying relative expression. ^*^P < 0.05; ^**^P < 0.01

We aimed to examine the heat damage status of different types of spermatogenic cells. Then, we selected the spermatogenic stem cell marker PLZF and spermatocyte marker BOULE for Western blot analysis (Fig. 3a). The results suggested that there was no difference in PLZF protein expression levels among the three groups (Fig. 3b). In contrast, the BOULE protein expression level in the HS group decreased significantly. Then, BOULE protein levels in the MT group showed a significant rebound compared to the HS group (Fig. 3b). Therefore, we selected the BOULE protein to verify this finding and performed immunofluorescence co-staining with TUNEL (Fig. 3c). Consistently, more than 60% of the TUNEL-positive localization in the visual field coincided with the BOULE protein localization (Fig. 3d). These results suggested that the number of spermatocytes was significantly rescued in the MT group.

It is also noteworthy that the survival rate of the round spermatozoa and the elongated spermatozoa differed significantly under heat stress. While the number of elongated spermatozoa did not change obviously, we observed that the number of round spermatozoa in the HS group was minimal by H&E staining (Fig. 3e). Therefore, we speculate that heat stress mainly affected the number of round spermatozoa. Toluidine blue staining further confirmed this result (Fig. 3e). The number of elongated spermatozoa (long-tailed arrow) was not significantly different from that in the HS and MT groups (Fig. 3f). However, the number of round spermatozoa (short-tailed indicator) was significantly reduced in the HS group, confirming the above results (Fig. 3g). Together, these results indicated that heat stress has significantly different effects on different types of spermatogenic cells. Heat stress mainly reduces the number of spermatocytes and round spermatozoa by inducing cell apoptosis. Melatonin pretreatment significantly inhibited cell apoptosis, protected spermatocytes and round spermatozoa from heat stress damage, and maintained the number of spermatogenic cells.

### Analysis of transcriptome sequencing (RNA-seq) results

**Fig. 4:**
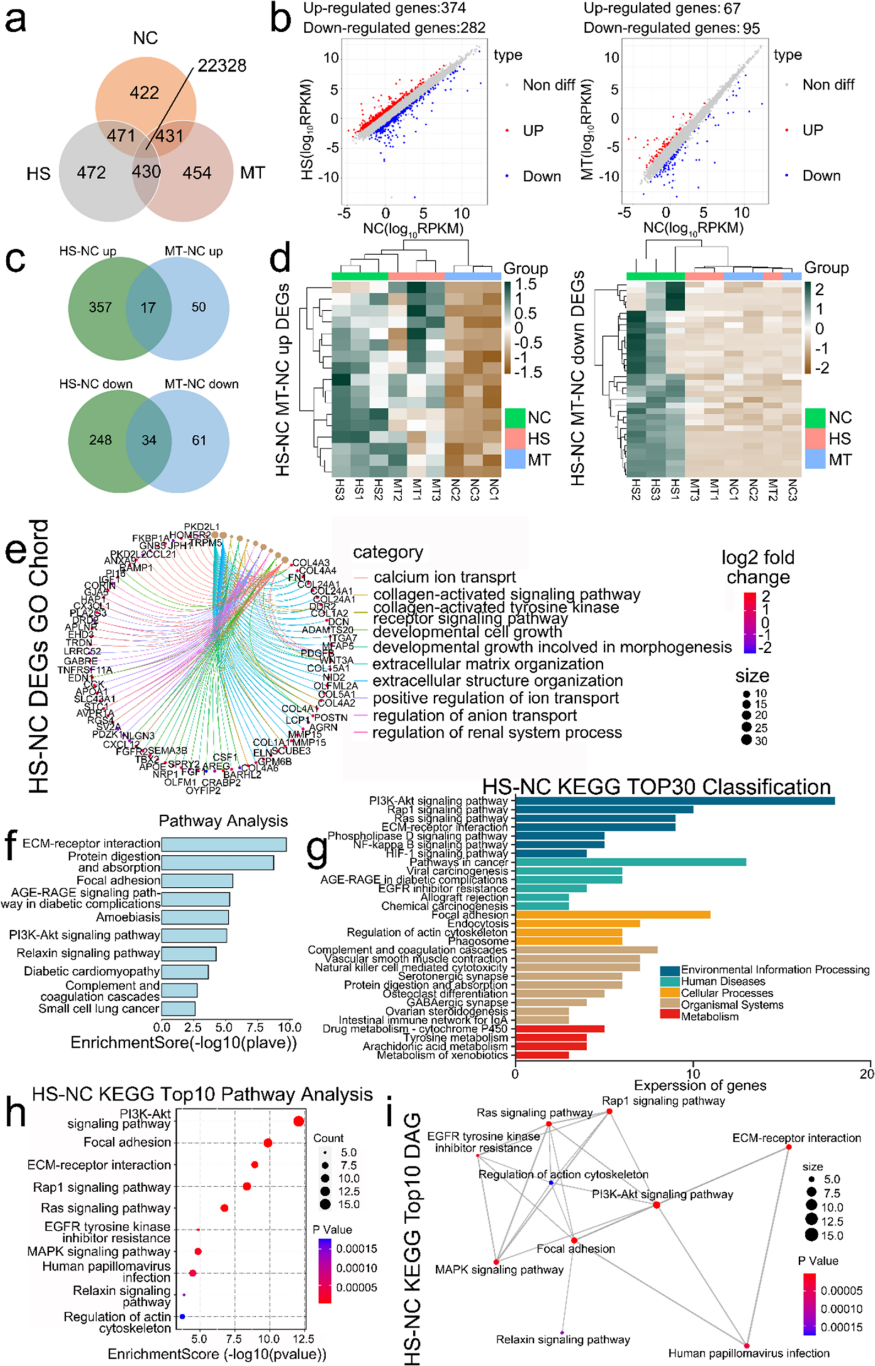
Analysis of three groups of transcriptome sequencing results. **a** Venn diagram of enriched genes in the three groups. **b** Left. Scatter plot of gene expression in the heat stress group. Red: upregulated genes; blue: downregulated genes; Right. Scatter plot of melatonin group gene expression. Red: upregulated genes; blue: downregulated genes. **c** Above. HS vs. NC | MT vs. NC UP Genes Venn; Below. HS vs. NC | MT vs. NC DOWN Genes Venn. **d** Left. Heatmap of Fig. 4c. above; Right. Heatmap of Fig. 4c. below. **e** HS vs. NC: Gene-GO terms | Gene-KEGG pathways. **f** KEGG signaling pathways of HS vs. NC DEGs. **g** HS vs. NC top 30 KEGG pathways. **h** Selected HS vs. NC KEGG 10 pathway analyses. **i** HS vs. NC DAG of KEGG 10 selected

We attempted to explore the differences in gene expression of testicular tissue cells between the HS and MT groups by RNA-Seq analysis of testicular tissue cells collected from dairy goats with experimental treatment. First, we focused on the different gene expression (DEGs) in the HS group compared to the NC group (log2FC > 1 and *P* value < 0.05 between them; Fig. 4b), and plotted the Venn diagram of genes in the three groups (Fig. 4a). Next, we analyzed the DEGs between the groups by screening the differential genes for transcriptional changes in both directions in the HS and MT groups compared to the NC group (17 up vs. 34 down; Fig. 4c). Then, we performed cluster analysis (Fig. 4d). At the gene expression level, melatonin pretreatment significantly altered gene expression changes under heat stress, more closely resembling the NC group. We took the top 3000 DEGs with log2FC > 1 corrected *P* value ranking in the HS and NC groups for GO and KEGG correlation network graph analysis to explore which terms were mainly associated with HS-induced DEGs (Fig. 4e) and then plotted KEGG graphs (Fig. 4f). The results showed that DEGs were primarily enriched in the phosphoinositide 3-kinase (PI3K)/protein kinase B (AKT) and extracellular matrix (ECM) signaling pathways.

Next, we performed KEGG pathway enrichment using all DEGs between the HS and NC groups to investigate what mechanisms further lead to differential gene expression by affecting the overall level of heat stress. The top 30 KEGG enrichment pathways were classified according to *P* value with FDR screening (Fig. 4g). In addition, we screened 10 signaling pathways based on objectives and cell phenotype in our study (Fig. 4h). The results of the 10 KEGG pathways were used for DAG plotting (Fig. 4i). We finally selected the most significant and relevant the phosphoinositide 3-kinase/protein kinase B (PI3K/AKT) signaling pathway and rat sarcoma protein (RAS) signaling pathway for the next step of validation.

### Melatonin inhibits the HS-promoted PI3K/AKT signaling pathway and RAS signaling pathway

**Fig. 5:**
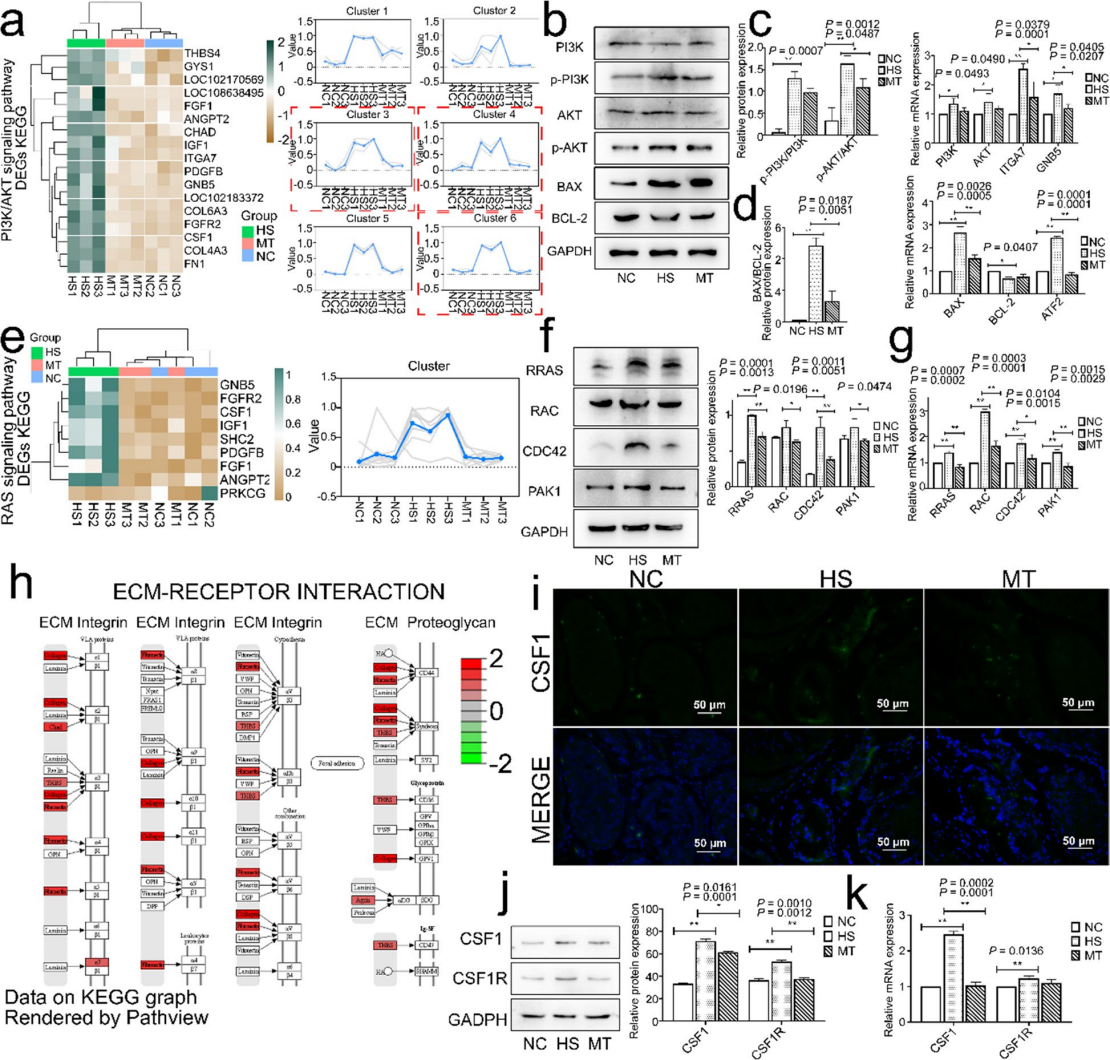
Melatonin inhibits both the RAS and PI3K/AKT signaling pathways activated by heat stress and reduces the expression levels of CSF1 and CSF1R. **a** Left. Heatmap of PI3K/AKT signaling pathway DEGs clustering; Right. Heatmap trend. **b** PI3K, p-PI3K, AKT, p-AKT, BAX, and BCL-2 protein expression levels. **c** Left. p-PI3K/PI3K, p-AKT/AKT quantification; Right. PI3K, AKT, ITGA7, and GNB5 mRNA expression levels. **d** Left. BAX/BCL-2 protein quantification; Right. BAX, BCL-2, ATF2 mRNA expression levels. **e** Left. Heatmap of RAS signaling pathway DEGs clustering; Right. Heatmap trend. **f** Left. RRAS, RAC, CDC42, and PAK1 protein expression levels; Right. Grayscale value analysis. **g** RRAS, RAC1, and PAK1 mRNA expression levels. **h** Expression profile of ECM KEGG. **i** CSF1 immunofluorescence staining. Green fluorescence, CSF1 localization. Blue fluorescence, localization of Hoechst 33342 dye (Scale bar: 50 μm). **j** Left. CSF1 and CSF1R protein expression levels; Right. Grayscale value analysis. **k** CSF1 and CSF1R mRNA expression levels. Mean grayscale values quantify relative expression. ^*^P < 0.05; ^**^P < 0.01

We used the 18 enriched DEGs in the PI3K/AKT signaling pathway to draw a clustered heatmap to validate the RNA-Seq analysis results (Fig. 5a) and then grouped every three genes with similar functions to plot a change trend chaperone (Fig. 5a). The related genes contained within clusters 3, 4, and 6, which were significantly and stably upregulated in the HS group and inhibited considerably by melatonin pretreatment, were selected for validation. Western blot results suggested that the HS group’s p-PI3K and p-AKT levels were significantly upregulated (Fig. 5b; c). The RT–qPCR results were consistent with this finding. The mRNA levels of PI3K, AKT, and the related membrane proteins ITGA7 and GNB5 were significantly upregulated between the HS and NC groups (Fig. 5c). The use of melatonin reduced the ratio of p-PI3K/PI3K and p-AKT/AKT (Fig. 5c) and inhibited the level of activating molecules of the PI3K/AKT signaling pathway. The results suggested that the ratio of BAX/BCL-2 was significantly upregulated in the HS group compared to the NC group (Fig. 5d) by using RT–qPCR and Western blotting to detect downstream BAX and BCL-2 proteins. Additionally, melatonin treatment significantly downregulated BAX/BCL-2, particularly increasing the level of BCL-2 protein (Fig. 5b; d). Thus, melatonin treatment reduced the level of p-AKT molecules not attributed to the execution of apoptosis. Together, melatonin pretreatment mitigated apoptotic signaling in germ cells in response to heat stress by inhibiting the PI3K/AKT signaling pathway.

The activation of the RAS signaling pathway is closely associated with the upregulation of p-AKT levels (Tenzer A, 2001). The trend changes of the RAS signaling pathway in RNA-seq analysis was similar to that of the PI3K/AKT signaling pathway (Fig. 5e). Western blot analysis showed that the protein levels of RRAS, RAC, CDC42, and PAK1 were significantly upregulated in the HS group (Fig. 5f), consistent with the clustering heatmap trend and mRNA expression levels (Fig. 5e; g). The binding level of RAS to GTP molecules increases under heat stress, which responds to extracellular stimuli and helps cells resist stress factors while activating the downstream PI3K/AKT signaling pathway and inducing p-AKT levels. In addition to the changes in the relevant signaling pathways, we also focused on the highly significant changes in the ECM components (Fig. 5h). We selected the colony-stimulating factor CSF1 and its receptor CSF1R for validation according to our experimental approach and study objectives. The activation of CSF1 suggests infiltration of immune cells such as macrophages and clearance and repair of damaged cells (Sehgal A, 2021). Experiments were first performed with immunofluorescence staining, which showed that melatonin pretreatment significantly reduced HS-induced CSF1 tissue infiltration (Fig. 5i). Heat stress significantly increased the mRNA and protein expression levels of CSF1 and CSF1R (Fig. 5j; k). In contrast, melatonin pretreatment inhibited their protein levels (Fig. 5j).

Together, these results suggested that heat stress significantly activated the RAS and PI3K/AKT signaling pathways in testicular cells, upregulated p-AKT levels, increased downstream apoptotic proteins, and induced apoptosis. Additionally, the increased colony-stimulating factor CSF1 and its receptor suggested an inflammatory response occurring locally in the testis and activating the body’s immune response. In contrast, melatonin pretreatment significantly inhibited these two signaling pathways, mainly by suppressing p-AKT levels, decreasing the apoptosis-directed BAX/BCL-2 ratio and infiltration level of CSF1, reducing tissue inflammation signaling, and protecting testicular tissue from heat stress damage.

### Validation of damage to different types of spermatogenic cells and melatonin restores local melatonin receptor levels inhibited by HS

**Fig. 6:**
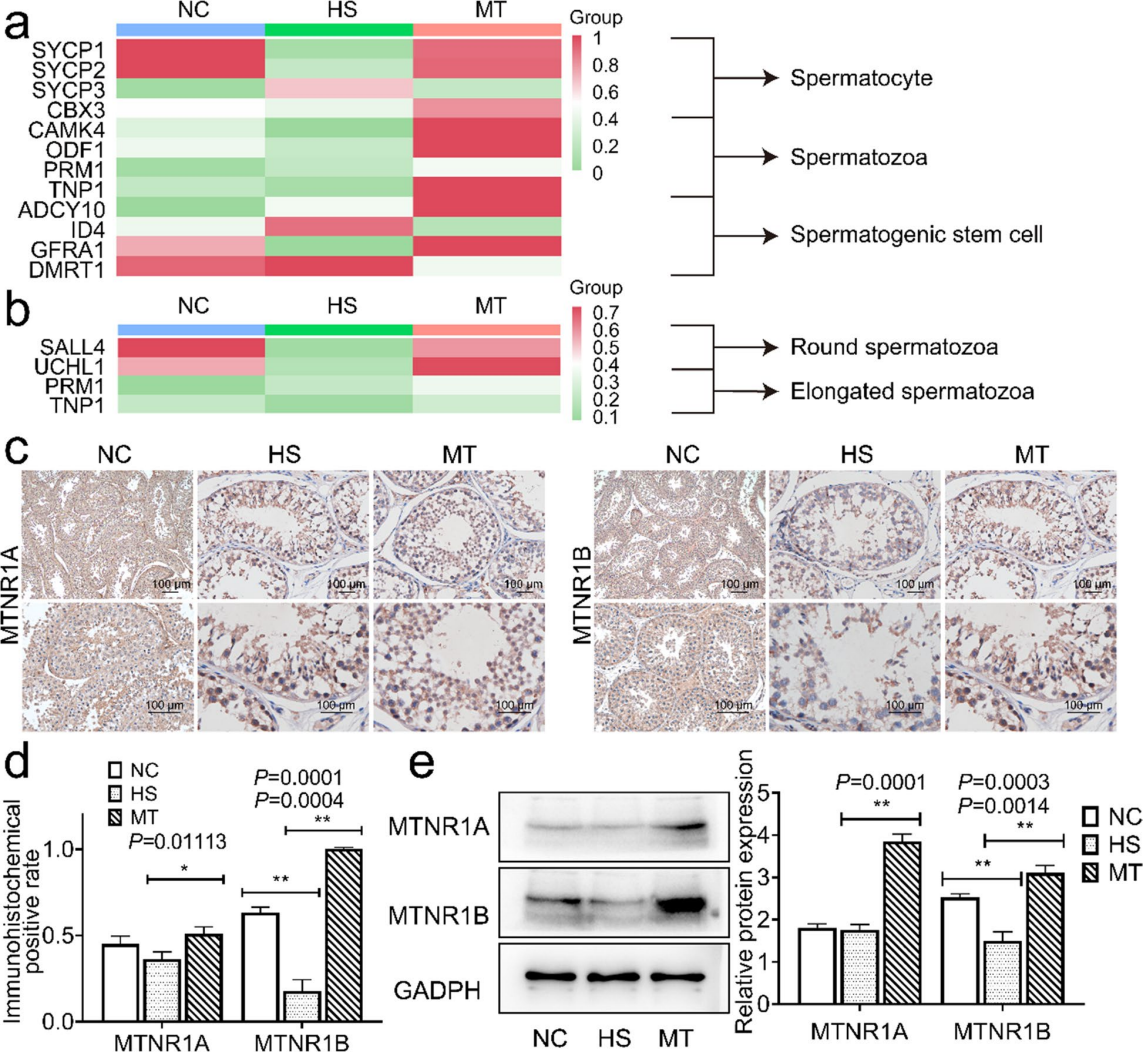
Validation of damage in spermatogenic cells and melatonin activates HS-suppressed melatonin receptor type 1B expression levels. **a** Heatmap of spermatocyte, spermatogenic stem cell, and spermatozoa markers. **b** Heatmap of round spermatozoa and elongated spermatozoa. **c.** Left. MTNR1A IHC localization; brownish-yellow particles (Scale bar: 100 μm); Right. MTNR1B IHC localization; brownish-yellow particles (Scale bar: 100 μm). **d** Positive rate statistics of Fig. 6c. **e** Left. MTNR1A and MTNR1B protein expression; Right. Grayscale value quantification. Mean grayscale values quantifying relative expression. ^*^P < 0.05; ^**^P < 0.01

First, our data link the comparison of damage to different types of spermatogenic cells to RNA-seq by analyzing the gene expression levels of various cellular markers, such as SYCP1, CAMK4, and ODF1. The raw expression of varying germ cell markers in representative samples of each group was selected to make a heatmap (Fig. 6a). The heatmap showed that markers of spermatogenic stem cells, such as DMRT1 expression, were more stable in each group, with no significant differences or inconsistent trends (Fig. 6a). In contrast, markers of spermatocytes, such as SYCP1 and SYCP3, were significantly suppressed in the HS group and restored in the MT group (Fig. 6a). Distinguishing the expression levels of the spermatozoa, we found that the expression levels of round spermatozoa markers, such as SALL4 and UCHL1, were significantly suppressed in the HS group and recovered in the MT group. In contrast, the expression levels of elongated spermatozoa markers, such as PRM1 and TNP1, did not change significantly in the different groups (Fig. 6b). These results demonstrate that heat stress mainly affects spermatocytes and spermatozoa and are consistent with the results in section 3.

In the final part of the study, we examined the levels of melatonin receptors 1A and 1B in testicular tissue to investigate how melatonin protects against heat stress. There was little change in melatonin receptor 1A expression between the HS and NC groups (Fig. 6e). However, heat stress significantly inhibited the protein expression level of melatonin receptor 1B (Fig. 6e), which indicated that the animals were in a physiological state of endogenous melatonin resistance under HS. Immunohistochemistry (IHC) results confirmed these results (Fig. 6c). The results in this section confirm the potent effect of heat stress on spermatocytes and round spermatozoa and indicate that melatonin can significantly activate the level of melatonin receptor 1B and thus alleviate the endogenous melatonin resistance of the animal organism under the influence of heat stress.

## Discussion

Seasonal heat stress significantly impairs livestock reproductive capacity in animal husbandry. Protecting male performance during summer has long been a concern. Goats have a higher tolerance under HS than other animals (Thornton P, 2021), and physiological indicators in goats are quite normal under these environmental conditions. However, this fact does not mean that their reproductive capacity isn’t impaired. In this experiment, we explored the use of melatonin as a potential therapeutic approach for preventing the apoptosis of HS-exposed spermatogenic cells and ameliorating decreased semen quality; we used male dairy goats to establish a model of natural heat stress in order to elucidate the possible mechanisms underlying the protective effects of melatonin. Our results suggest that melatonin can alleviate spermatocyte apoptosis, protect the structure of the spermatogenic tubules, and alleviate testicular heat damage in dairy goats by inhibiting the PI3K/AKT signaling pathway, ultimately improving semen quality.

Physiological melatonin concentrations are influenced by circadian rhythms, 80% of melatonin is synthesized at night, and serum melatonin concentration is 100–500 pg/mL in goats. Daytime serum melatonin concentration below 30 pg/mL (Chesneau D, 2017). Due to the principle of drug metabolism, melatonin plasma concentrations in the MT group are not constant over a 10-day period. We assumed that plasma concentrations of melatonin peak at 6–8 ng/mL based on other studies (Pool KR, 2020). The experiment monitored semen quality over a spermatogenic cycle. Previous studies have shown that oxidative stress triggered by multiple factors, including hyperthermia, is significantly associated with sperm dysfunction (Swain et al., 2020), and this was clear in our experiments. Goats exhibited a significant decrease in sperm quality and fertilization ability after exposure to heat stress. It is reasonable to assume that even if the fertilization process is completed, the rates of miscarriage, embryo malformation, and inferior embryo development due to low sperm quality will be significantly increased. Melatonin can substantially protect against the low semen quality caused by HS, protect sperm structure and function, and promote semen recovery from damage and it can be used as a drug to prevent the effects of HS in male dairy goats. Whether melatonin can improve the breeding rate by improving semen quality needs further experimental exploration.

It is well documented that melatonin can be produced by mitochondria (Suofu Y, 2017), and accumulate in the mitochondrial matrix, maximizing its antioxidant and modulatory activity (Pool KR, 2021; Tan DX, 2016). Being an amphiphilic molecule, melatonin is able to penetrate all cellular compartments, with the highest concentrations detected in mitochondria (Venegas et al., 2012). It is able to balance the mitochondrial membrane potential (Jauhari A, 2020) and is classified as a mitochondria-targeting antioxidant (Tan DX, 2020). Research shows that MT1 was found to have a variable distribution on the sperm head, depending on the spermatozoa and the individual male, whereas MT2 seems to be more restricted to the post-acrosomal and neck regions (Fernández-Alegre E, 2022), where are enriched with mitochondria. To address this point in more depth, in addition to exploring the mitochondrial morphology of HS and MT sperm, the link between them can be determined by comparing the proportion of different types of malformed sperm under different conditions, whether the typical heat-stressed malformed sperm is head, neck or tail malformed and which type of sperm is significantly reduced following melatonin treatment. Thus, we hypothesize that most of the positive effects of melatonin on sperm structure and function occur at the mitochondrial level, and further experiments are needed to explore.

Testicular tissue damage is the leading cause of impaired fertility. Our study shows that melatonin protects against testicular under HS by reducing oxidative stress, consistent with the results that melatonin protects against cadmium-induced cell stress in the testicular UPR (Ji YL, 2012). In this experiment, the effect of heat stress on spermatogenic cells was focused on spermatocytes and round spermatozoa. The survival rates of these two types of cells were significantly improved in the MT group, indicating that melatonin could protect these cells and maintain some of their spermatogenic functions during heat stress. At the individual level, it has been shown that the processes of spermatocyte differentiation and round spermatozoa metaplasia during spermatogenesis are dependent on androgen receptor levels (Wang RS, 2009). There is a link between the protective effect of melatonin on spermatogenic cells and its modulatory effect on testosterone. In addition, the data suggested that some spermatogenic stem cell markers, such as DMRT1 and ID4, were increased in the HS group. It is reasonable to hypothesize that HS may inhibit spermatogenic stem cell differentiation and promote proliferation, leading to upregulation of the expression of their markers.

The GO and KEGG analyses suggested that the most significant changes occurred in the PI3K/AKT signaling pathway, which was associated with the most differentially expressed genes. The PI3K/AKT pathway is a common signaling pathway that has been shown to be related to organismal immune responses in oxidative stress-related studies (Zhu H, 2022; Wang SF, 2021). Oxidative stress plays an essential role in HS-induced cell damage (Huang Y, 2022). ROS can act directly on AKT to activate the process of DNA damage repair (Zhou J, 2022; Chang KF, 2020). p-AKT induces the expression of the downstream apoptotic proteins BAX and BCL-2 to activate apoptotic responses (Yang et al., 2019b, 2020). The ratio of BAX/BCL-2 determines the course of the apoptotic response (Chen R, 2021). Our data suggested that melatonin inhibited the apoptotic process mainly by decreasing the p-AKT level and increasing BCL-2 expression. In addition, the BAX level was not significantly reduced in the MT group compared to the HS group, suggesting that the reduced apoptotic response in the MT group was not exclusively attributable to the molecular function of p-AKT.

It has been reported that RAS signaling, which participates in various pathway networks, is activated in response to oxidative stress (Ney GM, 2021) and extracellular stimulation (Cuevas-Navarro A, 2021); RAS signaling activates the downstream ERK and PI3K/AKT signaling pathways, increases p-AKT expression (Li L, 2009), and helps cells stabilize protein structures (Tsai WB, 2016) and avoid apoptosis (Tenzer A, 2001). Our analysis and experimental validation showed that RAS pathway activation was closely associated with HS-induced oxidative stress and p-AKT upregulation.

The heat shock and inflammatory responses in an organism are usually accompanied by the formation and degradation of the extracellular matrix (Shi P, 2022). The changes in the extracellular matrix composition observed in the sequencing data are also of interest. Melatonin significantly directly or indirectly inhibited the levels of CSF1 and CSF1R, confirming its role in alleviating testicular thermal injury and the immune response in the organism. The HS-induced changes in the components of the extracellular matrix are incredibly significant and widespread, and the mechanisms underlying many molecular-level changes and interactions remain to be explored.

In the final part of the study, we found that under HS, the male reproductive system is in a physiological state of endogenous melatonin resistance, which is attributed to changes in the levels of melatonin receptor 1B. In our study, it is clear that there is a link between the improvement in the local levels of melatonin, a reproductive regulatory hormone, in the testis and changes in melatonin receptor levels. The mechanism underlying this interaction still needs to be further explored. Additionally, the effect of HS on the physiological state of the organism is comprehensive and widespread, and effects on MT2 may also be observed in other tissues.

## Conclusion

Our results suggested that melatonin alleviates testicular tissue damage in male dairy goats under HS and protects spermatocytes and round spermatozoa, which ultimately manifests clinically as improved semen quality. We screened some critical genes involved in melatonin repair of the HS effect with key pathways by RNA-Seq. These results provide an important reference for subsequent studies on the molecular mechanisms by which melatonin changes male reproductive processes under HS. In addition, our results provide a connection for using exogenous melatonin in production to prevent and control the adverse effects of HS on breeding and reproduction.

## Materials and methods

### Natural heat stress and melatonin treatment

Twelve caprine (*Capra aegagrus* hircus) of Saanen male breed aged 1 year and 6 months, imported from New Zealand, were placed in a 25*30 m^2^ house, with free feeding and watering, from the Red Star Antelope Eco-Economic Recycling Park in Fuping County, Weinan City, Shaanxi Province. During the 10-day molding period, the roof was removed daily from 12:00 to 16:00 to expose the goats to sunlight. The ambient temperature (Temperature, T, °C) and air humidity (AH, %) were measured at the beginning and end of the time, and then the THI was calculated by the following equation (Ravagnolo O, 2000). In the MT group, the 80 μg/kg melatonin injection was administered subcutaneously daily during the modeling period. Melatonin powder (Dean, Zhengzhou, China) was dissolved in anhydrous ethanol and diluted in a gradient of distilled water to an injection concentration of 10 mg/mL with ethanol content < 1%. Selection of melatonin dosage referenced from a study using subcutaneous melatonin implants (Yang CH, 2020; Yang et al., 2019a, b), and the application method was derived from studies in male goats (Quillinan NP, 2014).

$$THI=\left({1.8}^{\ast }\ T+32\right)-\left[{\left(0.55-{0.0055}^{\ast }\ AH\right)}^{\ast }\ \left({1.8}^{\ast }\ T-26\right)\right]$$

All goat experiments were approved by the guidelines of the Northwest A&F University Animal Care and Use Committee (approval number: GB/T45785–2021).

### Semen collection and identification of quality

Semen was collected once every 3 days with the artificial vagina (10 ejaculations/month * 2 months = 20 ejaculations/animal) for 2 months or 61 days. One hundred microliters were taken from the semen samples and diluted in 40X. The Mylan Animal Sperm Analyzer (Songjing, China) was used to analyze sperm density, sperm motility, sperm viability, sperm foregone, and effective sperm count. Spermatozoa were graded by calculating the average rate of sperm movement: fast progressive movement (VAP ≥ 25 μm/s) at 37 °C was considered as grade A spermatozoa; slow or slow progressive movement (25 > VAP > 0 μm/s) was considered as grade B spermatozoa; non-progressive movement (VAP = 0 μm/s) was considered as grade C spermatozoa. The same volume of samples was taken and diluted to 100 X. The following morphological identifications were performed using light microscopy: sperm malformation rate, sperm acrosome integrity rate, and sperm membrane integrity rate. Morphological identification was performed by counting 500 spermatozoa per sample for each index.

### Testicular tissue collection and histopathological examination (HE) staining

Testicular sampling was performed on experimental animals 6 days after the experimental treatment. The testes were anesthetized by intramuscular injection of a 30 mg/kg dosage of sodium pentobarbital. The testes were removed, cut into appropriate-sized tissue pieces, and placed into 4% PFA and liquid nitrogen tanks. Paraffin sections were made and stained with hematoxylin and eosin.

### Mid-end dUTP nick end segment labeling (TUNEL) staining

Two-micrometer-thick white slices were made, dewaxed, rehydrated, repaired with 10% FBS, stained using the TUNEL one-step assay kit (Biyuntian, Shanghai, China), sealed with Hoechst 33342 nucleation agent and anti-fluorescence quencher, and photographed with the EVOM™M5000 imager (Thermo Fisher Scientific, USA).

### Toluidine blue staining

Two-micrometer-thick white slices were made, dewaxed, rehydrated. Then add toluidine blue staining solution dropwise for 15 min. Distilled water wash 2 times, each time 5 min. 95% alcohol differentiation, mast cells with deep blue-purple, the background is light blue or colorless, microscopic observation of coloring. Finally, put into xylene and use neutral resin to seal the film. Ten fields of view were selected for each group of staining for elongated spermatozoa and round spermatozoa.

### Immunofluorescence staining

Two-micrometer-thick white slices were made, dewaxed, rehydrated, repaired with 10% FBS, incubated with primary coupled antibodies CSF1(BBI life sciences, Shanghai, China) for 12 hours at 4 °C, and then, incubated with horseradish peroxidase (HRP)-conjugated secondary antibodies for 1 hour at 37 °C. Hoechst33342 nucleating agent was blocked with anti-fluorescence quencher, and photographs were taken using an EVOM™M5000 imager (Thermo Fisher Scientific, USA) to take photographs.

### Immunofluorescence co-staining

The section processing and antibody incubation procedures were the same as in the immunofluorescence staining section. The primary coupled antibodies were BOULE (Abcam, USA). Then using the TUNEL one-step assay kit (Biyuntian, Shanghai, China). Hoechst 33342 nucleation agent was blocked with anti-fluorescence quencher and photographs were taken using an EVOM™M5000 imager (Thermo Fisher Scientific, USA) to take photographs.

### Serum collection and ELISA tests

Blood samples were collected from the jugular vein of goats into disposable negative pressure PRP vacuum tubes, placed at 4 °C for 1 h, and centrifuged at 3000 rpm for 15 min to collect the serum. Testosterone was measured using a goat testosterone ELISA kit (Maiman, Jiangsu, China). An ELX808 absorbance reader was used to measure the OD value at 450 nm.

### Western blot

Protein lysis and extraction were performed using RIPA lysis buffer. Protein extraction samples were subjected to 12% sodium dodecyl sulfate–polyacrylamide gel electrophoresis and transferred to PVDF membranes, which were incubated with primary coupled antibodies (Table. S1) for 12 h at 4 °C. Then, the PVDF membrane was incubated with horseradish peroxidase (HRP)-conjugated secondary antibody for 1 h at 37 °C. Gel imaging was performed in an iBright CL1500 imaging system (A44240, Thermo Fisher Scientific, USA), and grayscale values were analyzed using ImageJ (version 5.0, Bio-Rad, Hercules, CA, USA) and normalized to glyceraldehyde-3-phosphate dehydrogenase (GAPDH).

### RNA-seq and bioinformatics analysis

Whole-genome transcriptome sequencing was performed by Kang Test (Wuhan, China). Transcriptome sequencing was used to identify mRNA products between natural control differentially expressed, heat stress-treated, and melatonin-treated testis tissues collected from dairy goats. RNA preparation for transcriptome sequencing and library preparation was performed according to the manufacturer’s instructions. Used the edge R package (version 3.12.1) to identify genes that were differentially expressed between groups. A fold change cut-off of 2 and a *P* value cut-off of 0.05 was used to determine the statistical significance of gene expression differences. Gene Ontology (GO) analysis and Kyoto Encyclopedia of Genes and Genomes (KEGG) enrichment analysis of differentially expressed genes were both implemented by KOBAS software (version: 2.1.1) with a *P* value cut-off of 0.05 to determine the statistical significance of enrichment.

### RNA extraction and real-time quantitative PCR (RT–qPCR)

Samples were incubated with TRIzol reagent to extract total RNA. cDNA was synthesized using the All-in-One™ cDNA Synthesis Kit (FulenGen, Guangzhou, China). cDNA was subsequently synthesized using Taq SYBR green qPCR Premix (Applied Biosystems, Foster City, CA). The reaction volume was 20 μl, and the reaction conditions were set to 95 °C for 2 min, 95 °C for 10 s, and 60 °C for 30 s for 40 cycles, and 60 °C for 5 s. The primer sequences used are shown in Table. S2. Data analysis used the 2^^△△CT^ method. Normalized levels to glyceraldehyde-3-phosphate dehydrogenase (GAPDH).

### Statistical analysis

Values are expressed as the mean ± standard deviation (SD) with at least three independent experiments repeated. Data were tested for conformity to a normal distribution in SPSS 26.0, then analyzed and graphed using t-tests in GraphPad Prism 8.0.2 software. A *P* value < 0.05 was considered a statistically significant difference, and a *P* value < 0.01 was considered a highly significant difference.

## Supplementary Information

**Additional file 1: Table S1.** List of antibodies for Western Blot. **Table S2.** List of Primers for RT-qPCR. **Table S3.** Temperature and Air Humidity during Moulding. **Fig. S1.** Graphic abstract. Fig. S2 Editing Certificate. **Fig. S3.** Saanen dairy goat importation genealogy certificate. **Fig. S4.** Ethics approval.

## Data Availability

All data generated or analyzed during this study are included in this published article and its supplementary information files.
