# Supplementary material for: Melatonin alleviates heat stress-induced testicular damage in dairy goats by inhibiting the PI3K/AKT signaling pathway
Source: Stress Biol. 2022 Nov 14;2(1):47. doi: 10.1007/s44154-022-00068-9 (PMC10441922; doi:10.1007/s44154-022-00068-9)
Supplement: Supplementary file 1 — Additional file 1: Table S1. List of antibodies for Western Blot. Table S2. List of Primers for RT-qPCR. Table S3. Temperature and Air Humidity during Moulding. Fig. S1. Graphic abstract. Fig. S2 Editing Certificate. Fig. S3. Saanen dairy goat importation genealogy certificate. Fig. S4. Ethics approval. [file 44154_2022_68_MOESM1_ESM.docx]

**Supplementary Information**

**Table. S1**

Table. S1 List of antibodies for Western Blot

| Polyclonal antibody | Brands | Dilution proportion | Company |
| --- | --- | --- | --- |
| HSP70 | Abmart | 1:2000 | Shanghai, China |
| CASP 3 | Wanlei | 1:2000 | Shenyang, China |
| CASP 8 | Proteintech | 1:1000 | Wuhan, China |
| PLZF | Boster | 1:500 | Hubei, China |
| BOULE | Abcam | 1:2000 | Cambridge, UK |
| PI3K | Proteintech | 1:4000 | Wuhan, China |
| p-PI3K | Immunoway | 1:2000 | Plano, USA |
| AKT | Immunoway | 1:4000 | Plano, USA |
| p-AKT | Immunoway | 1:4000 | Plano, USA |
| BAX | Abmart | 1:2000 | Shanghai, China |
| BCL-2 | Abmart | 1:2000 | Shanghai, China |
| RRAS | Wanlei | 1:500 | Shenyang, China |
| RAC | Wanlei | 1:1000 | Shenyang, China |
| CDC42 | Wanlei | 1:500 | Shenyang, China |
| PAK1 | Wanlei | 1:500 | Shenyang, China |
| CSF1 | BBI life sciences | 1:500 | Shanghai, China |
| CSF1R | BBI life sciences | 1:500 | Shanghai, China |
| MTNR1A | Bioss | 1:1000 | Beijing, China |
| MTNR1B | Bioss | 1:1000 | Beijing, China |

**Table. S2**

Table. S2 List of Primers for RT-qPCR

| Gene | 5'->3' on plus strand | 5'->3' on minus strand | Product size (bp) |
| --- | --- | --- | --- |
| HSP70 | CGACGTGTCCATTCTGACG | GCTTGTTCTGGCTGATGTCCT | 158 |
| Caspase3 | CACGAAGCAGACGCAGAT | GGTTGTTGTCCCGAGTCAT | 101 |
| Caspase8 | GAGCACTACCTCTCCTACCGACAC | GTGTAGCGTGGTTCTGGCATCTG | 189 |
| PI3K | CACAGCGTAGGTTATGAA | TATTTCTGCTGCTTCATC | 201 |
| AKT | CCAAGTCCTTGCTCTCGGG | CTCCATGCTGTCGTCTTGGT | 251 |
| ITGA7 | AACTTGCTGCTCAGAGATGCT | CTTGAAGAATCCCATCCCACAG | 284 |
| GNB5 | GGGATTCCTTCACCACTA | CATTTATTATCCAAACCACC | 118 |
| ATF2 | AAGAGGTAGTCTGATTGGCTTAACT | CACAGGTCCTTGTATTGCCTGG | 120 |
| BAX | GCGCATTGGAGATGAATTGGA | GAGAGGAGGCCGTCCCAA | 297 |
| BCL-2 | CAACGGAGGCTGGGACG | GAGCAGTGCCTTCAGAGACA | 91 |
| RRAS | AACAAGGCGGATCTGGAAA | TGGTGGGAGGCACTGAAGG | 71 |
| RAC1 | GTCAAGAAGCGGAAGAGG | GCAAAGCAAACAAAGGGT | 84 |
| CDC42 | ATGCCTGGGACGATTCAC | CCTCACCTGGCTGCTCTA | 170 |
| PAK1 | ATCAGACTGCCCAAACAC | GGTCCAGAAAGAAGAGCC | 105 |
| CSF1 | TGACTAAAGGGAGGGAAGAA | CTGGACAGGGAAAGAGGC | 164 |
| CSF1R | CATCACGGAGTATTGTTGCT | CCTGCGGATGTATTTCTTTT | 151 |

**Table. S3**

Table. S3 Temperature and Air Humidity during Moulding

| Temperature, ℃ | | Air Humidity, % | |
| --- | --- | --- | --- |
| 12:00 | 16:00 | 12:00 | 16:00 |
| 31.5 | 33 | 49 | 48 |
| 32 | 33.5 | 48 | 48 |
| 32.5 | 34.5 | 50 | 49 |
| 32 | 34 | 49 | 48.5 |
| 32 | 33 | 48 | 48 |
| 32.5 | 34.5 | 49 | 48 |
| 34 | 35.5 | 49 | 47.5 |
| 34.5 | 36 | 49 | 47 |
| 33 | 34.5 | 49.5 | 48 |
| 32.5 | 33.5 | 49.5 | 49 |

**Fig. S1 Graphic abstract**


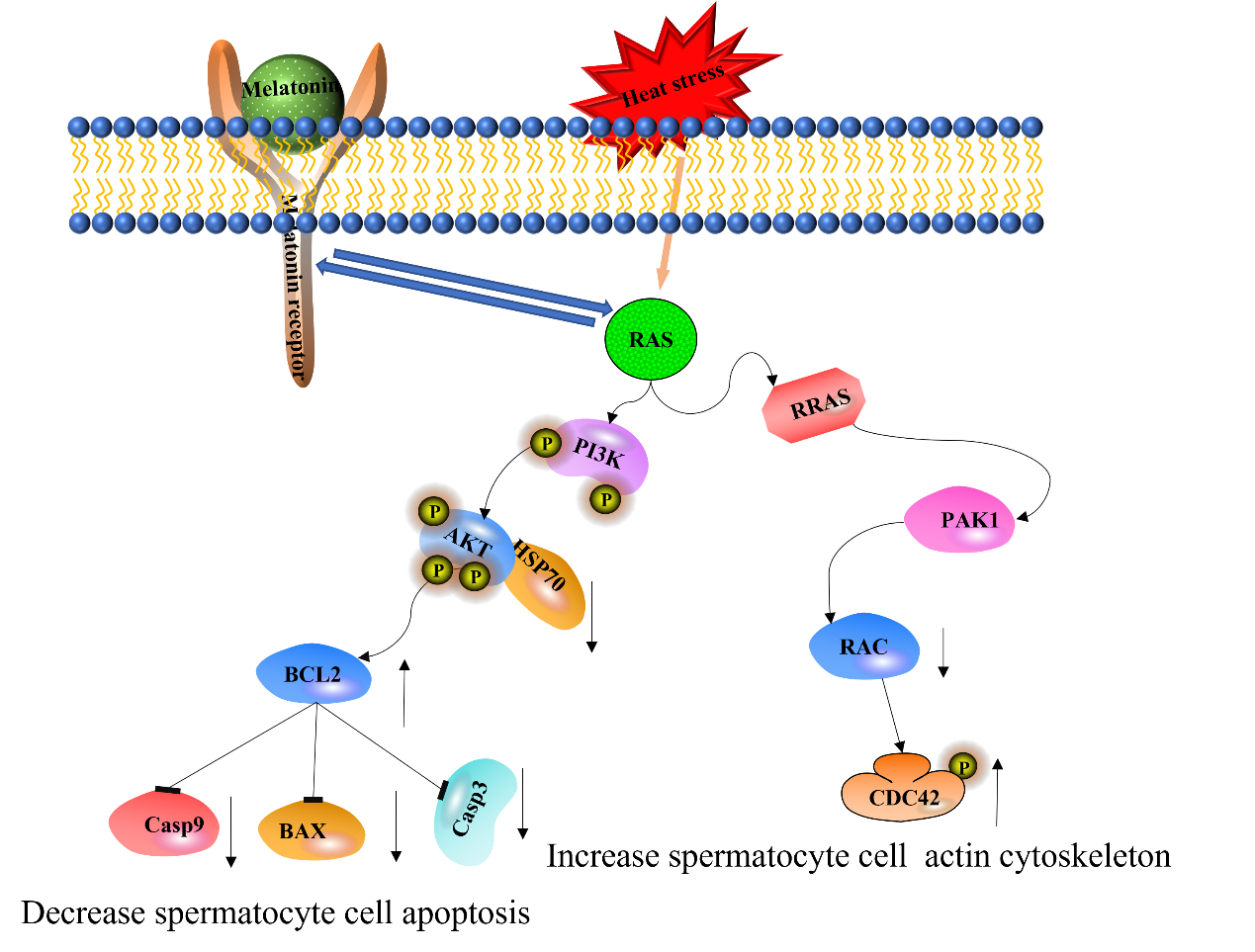
**Overview of the PI3K/AKT signaling pathway**

Underlying mechanism of MT inhibition of heat stress resistance via regulation of PI3K/AKT/RAS pathway. Melatonin increases Caspase9 dependent expression of BCL2, and inhibit BAX and Caspase3 protein activation. Leading to the decrease of apoptosis.

Melatonin modulated PI3K/AKT/RAS signaling pathway. Melatonin activated PI3K/AKT/RAS response pathway through binding to MT1B receptor. Leading to the increase of Increase spermatocyte cell actin cytoskeleton.

→ (Direct stimulatory modification); ┴ (Direct Inhibitory modification); →┤ (Multistep inhibitory modification); ↑ (Up regulate); ↓ (Down regulate); PI3K (Phosphoinositide 3-Kinase); AKT (protein kinase B); BAX (B-cell lymphoma-2-associated X protein)

**Fig. S2 Editing Certificate**

**
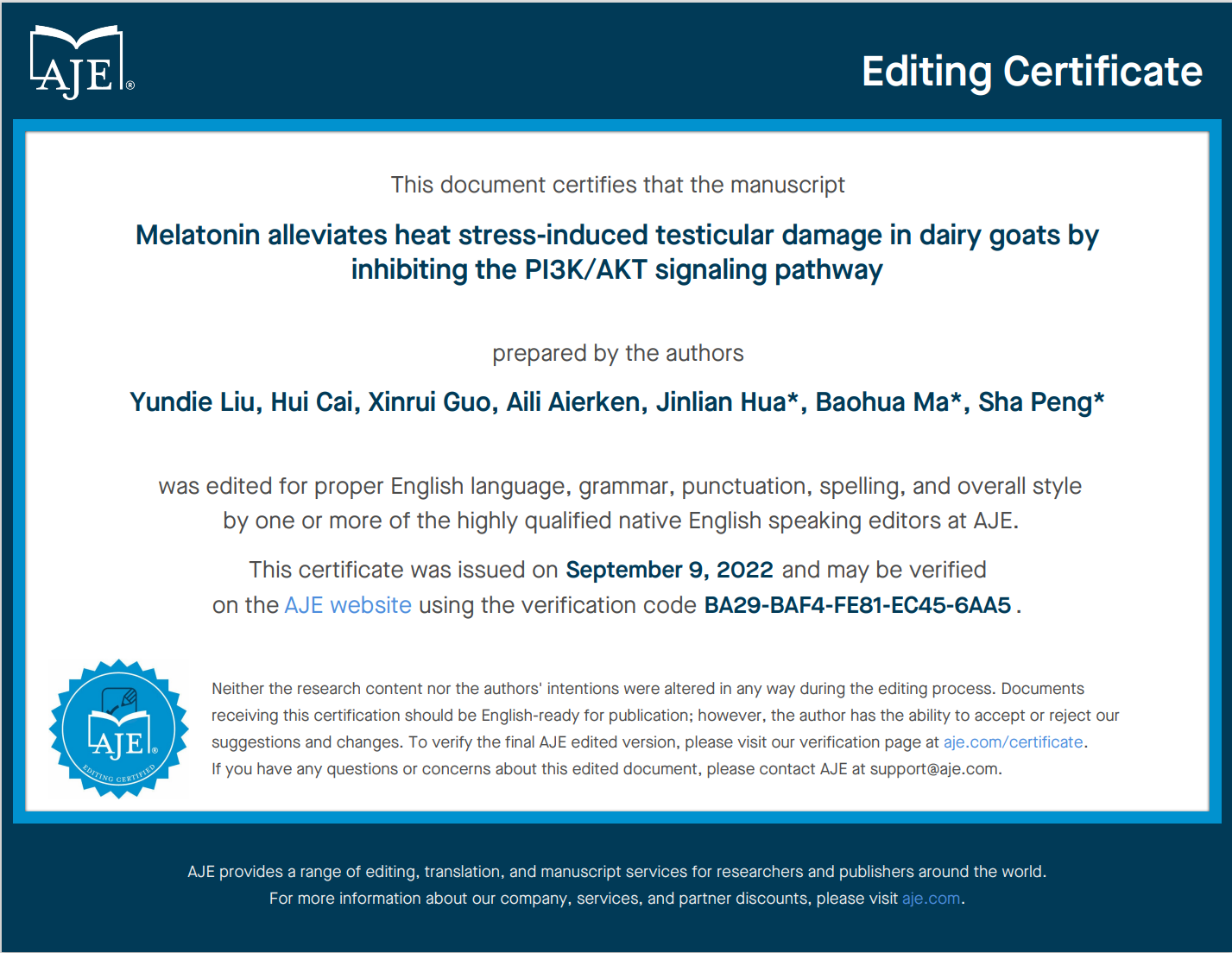
**

**Fig. S3 Saanen dairy goat importation genealogy certificate**

**
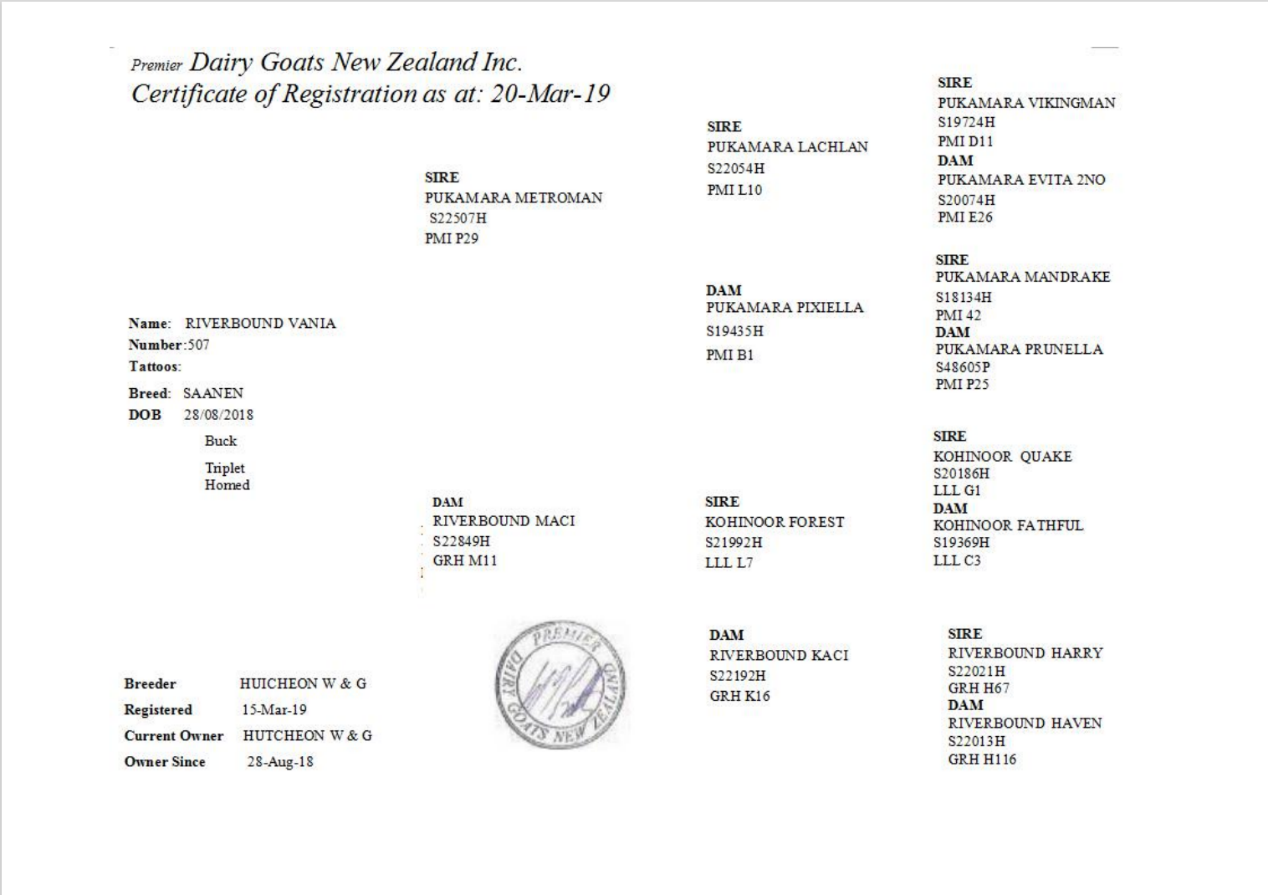
**

**Fig. S4 Ethics approval**

**
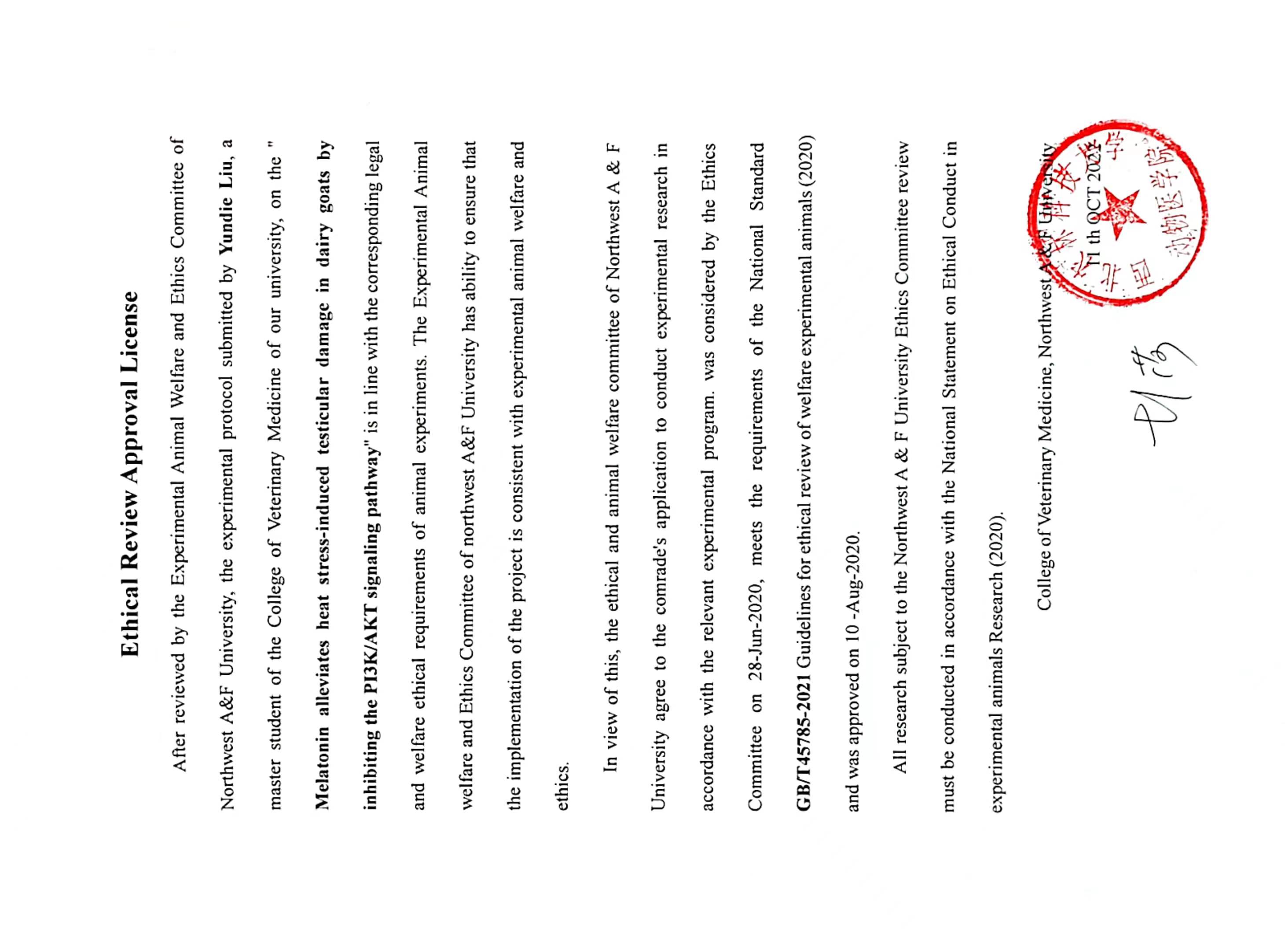
**
